# Gut microbiota from sigma-1 receptor knockout mice induces depression-like behaviors and modulates the cAMP/CREB/BDNF signaling pathway

**DOI:** 10.3389/fmicb.2023.1143648

**Published:** 2023-04-06

**Authors:** Jia-Hao Li, Jia-Li Liu, Xiu-Wen Li, Yi Liu, Jian-Zheng Yang, Li-Jian Chen, Kai-Kai Zhang, Xiao-Li Xie, Qi Wang

**Affiliations:** ^1^Guangzhou Key Laboratory of Forensic Multi-Omics for Precision Identification, School of Forensic Medicine, Southern Medical University, Guangzhou, China; ^2^Department of Toxicology, School of Public Health, Southern Medical University (Guangdong Provincial Key Laboratory of Tropical Disease Research), Guangzhou, Guangdong, China

**Keywords:** SIGMAR1, gut microbiota, depression-like behaviors, FMT, antibiotic, BDNF

## Abstract

**Introduction:**

Depression is a common mental disorder that affects approximately 350 million people worldwide. Much remains unknown about the molecular mechanisms underlying this complex disorder. Sigma-1 receptor (Sig-1R) is expressed at high levels in the central nervous system. Increasing evidence has demonstrated a close association between the Sig-1R and depression. Recently, research has suggested that the gut microbiota may play a crucial role in the development of depression.

**Methods:**

Male Sig-1R knockout (Sig-1R KO) and wild-type (WT) mice were used for this study. All transgenic mice were of a pure C57BL/6J background. Mice received a daily gavage of vancomycin (100 mg/kg), neomycin sulfate (200 mg/kg), metronidazole (200 mg/kg), and ampicillin (200 mg/kg) for one week to deplete gut microbiota. Fecal microbiota transplantation (FMT) was conducted to assess the effects of gut microbiota. Depression-like behaviors was evaluated by tail suspension test (TST), forced swimming test (FST) and sucrose preference test (SPT). Gut microbiota was analyzed by 16s rRNA and hippocampal transcriptome changes were assessed by RNA-seq.

**Results:**

We found that Sig-1R knockout induced depression-like behaviors in mice, including a significant reduction in immobility time and an increase in latency to immobility in the FST and TST, which was reversed upon clearance of gut microbiota with antibiotic treatment. Sig-1R knockout significantly altered the composition of the gut microbiota. At the genus level, the abundance of Alistipes, Alloprevotella, and Lleibacterium decreased significantly. Gut microbiota dysfunction and depression-like phenotypes in Sig-1R knockout mice could be reproduced through FMT experiments. Additionally, hippocampal RNA sequencing identified multiple KEGG pathways that are associated with depression. We also discovered that the cAMP/CREB/BDNF signaling pathway is inhibited in the Sig-1R KO group along with lower expression of neurotrophic factors including CTNF, TGF-α and NGF. Fecal bacteria transplantation from Sig-1R KO mice also inhibited cAMP/CREB/BDNF signaling pathway.

**Discussion:**

In our study, we found that the gut-brain axis may be a potential mechanism through which Sig-1R regulates depression-like behaviors. Our study provides new insights into the mechanisms by which Sig-1R regulates depression and further supports the concept of the gut-brain axis.

## 1. Introduction

Depression, also known as major depressive disorder (MDD), is a common mental disorder that affects approximately 350 million people worldwide. The causes of depression are thought to be multifaceted and may involve both genetic and environmental factors (Malhi and Mann, 2018). In the past few decades, various theories about the development of depression have been proposed, including the inflammation hypothesis, the neural circuits hypothesis, the neurotransmitter hypothesis, and the neurotrophic hypothesis (López-Figueroa et al., 2004; Duman and Monteggia, 2006; Fujimoto et al., 2008; Maes, 2008). The neurotrophic hypothesis suggests that brain-derived neurotrophic factor (BDNF) and other neurotrophic factors may be involved in the development of depression, and reductions in BDNF have been linked to atrophy of brain regions involved in emotion, such as the hippocampus (Castren and Rantamaki, 2010). Neurotrophic factor BDNF has also turned out to be significantly associated with depression in clinical patients and in extensive studies. In recent years, the neurotrophic hypothesis has become an important target of novel antidepressant drugs (Wang A. et al., 2021). Despite significant progress in our understanding of depression, much remains unknown about the molecular mechanisms underlying this complex disorder.

Sigma-1 receptor is a protein encoded by the SIGMAR1 gene. Sig-1R is expressed at high levels in the central nervous system and has been shown to have neuroprotective effects (Wu et al., 2021). Mutations in the SIGMAR1 gene have been linked to an increased risk of cognitive dysfunction and are strongly associated with Alzheimer’s disease (AD; Fehér et al., 2012). In addition, two studies have found that Sig-1R may be involved in the development of depression through its effects on neurotrophic and growth factor signaling pathways (Kishi et al., 2010; Mandelli et al., 2017; Stiernstromer et al., 2022). Selective serotonin reuptake inhibitors (SSRIs) have a moderate affinity for Sig-1Rs and the antidepressant effect of SSRIs may be mediated by Sig-1R (Smith and Su, 2017; Izumi et al., 2023). Efficacy of fluvoxamine treatment in improving psychotic symptoms and depressive symptoms is dose-dependently related to its binding to Sig-1Rs in the healthy human brain (Hashimoto, 2009). One potential mechanism through which Sig-1R may exert its neuroprotective effects is by promoting brain-derived neurotrophic factor (BDNF) expression. For example, Sig-1R agonists have been shown to reverse the downregulation of BDNF and improve symptoms of post-traumatic stress disorder (PTSD; Ji et al., 2017). Another Sig-1R agonist has been demonstrated to have neuroprotective effects in a mouse model of alpha-thalassemia X-linked intellectual disability (Yamaguchi et al., 2018). The brain’s BDNF protein was elevated by chronic antidepressant treatment, including SSRIs (Hashimoto, 2010). Activation of Sig-1R enhanced the conversion of pro-BDNF to mature BDNF, as well as the release of mature BDNF into the extracellular space (Fujimoto et al., 2012; Hashimoto, 2013). These pieces of evidence suggest that activation of the Sig-1R promotes chaperone activity, which influences BDNF secretion, exerting antidepressant-like effects. Despite these advances, the exact role of Sig-1R in the development of depression is not yet fully understood.

The relationship between gut microbiota and depression has garnered increasing attention in recent years, with a growing body of evidence demonstrating a significant association between the two. Studies have found that the gut microbiota of individuals with depression differs significantly from that of healthy individuals (Jiang et al., 2015; Aizawa et al., 2016). Moreover, transplanting the microbiota from depressed individuals into germ-free or antibiotic-treated mice has resulted in the induction of symptoms similar to depression (Kelly et al., 2016; Zheng et al., 2016; Li et al., 2019). These findings suggest that the gut microbiota may play a role in the pathogenesis of depression.

Despite the growing evidence linking Sig-1R to depression, the precise role of Sig-1R in this disorder remains unknown. To further understand the involvement of Sig-1R in depression, we conducted a study in which we compared depression-like behaviors in the wild-type (WT) and Sig-1R knockout (KO) groups. We also explored the role of gut microbiota in these behaviors through antibiotic treatment and fecal microbiota transplantation (FMT). Our results showed that Sig-1R knockout mice exhibited increased depression-like behaviors and significant changes in gut microbiota. The gut microbiota of Sig-1R knockout mice was sufficient to induce an increase in depression-like behaviors. These effects may be mediated by a reduction in neurotrophic factors through the cAMP/CREB/BDNF signaling pathway. Our study provides new insights into the mechanisms by which Sig-1R may contribute to the development of depression.

## 2. Materials and methods

### 2.1. Animals

During the study, the animals were kept in specific pathogen-free conditions in the laboratory animal center of Southern Medical University, with a 12-h light/dark cycle. Sig-1R knockout mice were obtained from Cyagen Biosciences. All transgenic mice were of a pure C57BL/6 J background. Sig-1R knockout mice and WT mice, identified by PCR genotyping (Supplementary Figure 1), were generated by breeding Sig-1R heterozygous mice with Sig-1R heterozygous mice. WT littermates of the same sex were used as controls in all experiments. Feces were collected from 10-week-old mice and behavioral tests were conducted. As for the collection of fresh fecal samples, mice were placed in a cage covered with autoclaved paper in a SPF environment and allowed to defecate freely between 9:00 and 11:00 AM. The fecal samples were collected using autoclaved RNA-free EP tubes, and then stored in −80°C until used. A total of three fecal samples were collected. From 7:00 PM onward, following euthanasia, serum and hippocampal tissue were collected for further analysis. Samples were rapidly frozen in liquid nitrogen and stored at −80°C until use.

### 2.2. Antibiotic treatment

**Figure 1:**
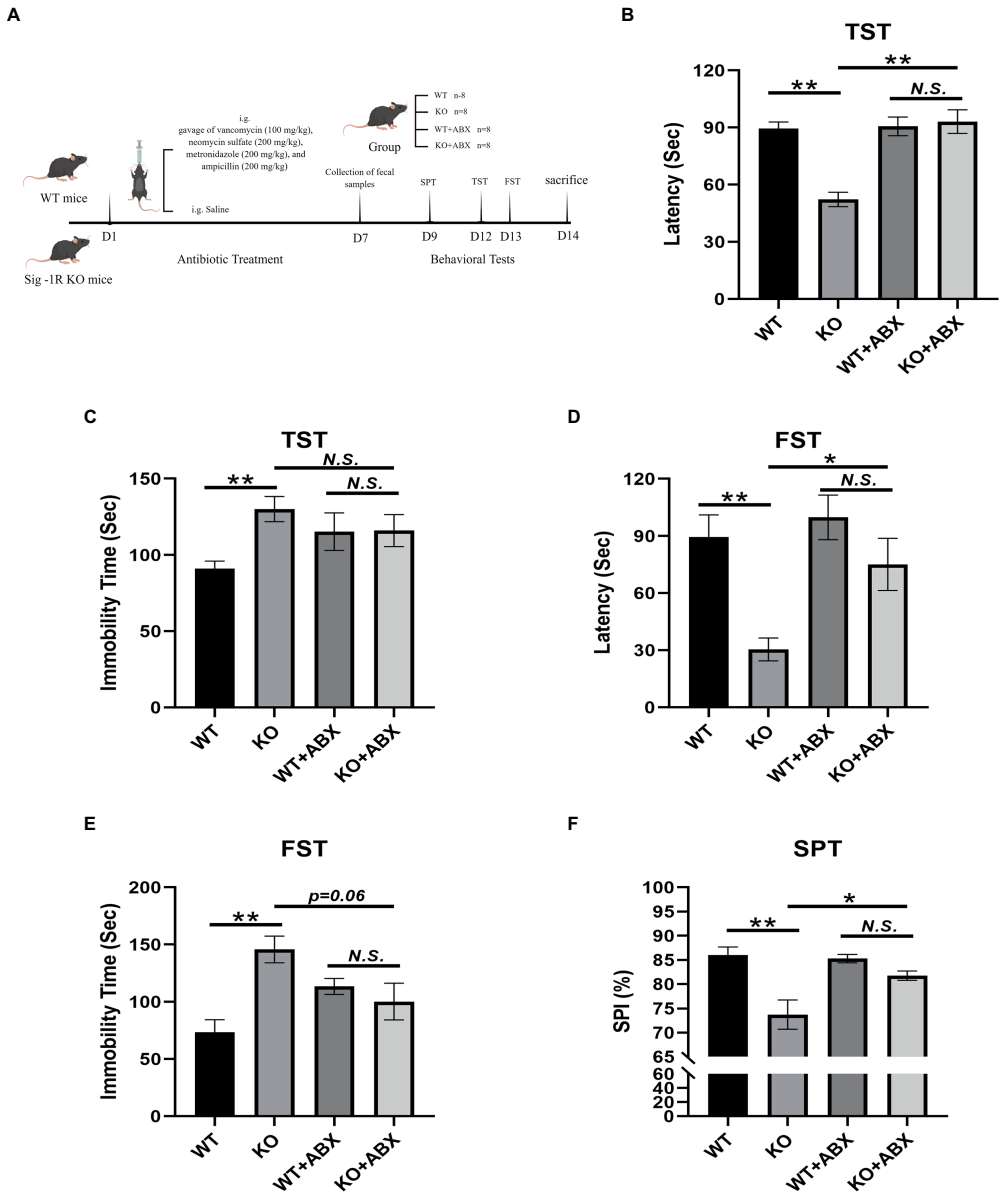
Antibiotic treatment reverses Sig-1R knockout-induced depression-like behaviors. **(A)** The ABX treatment flowchart by Figdraw. **(B)** Latency (s) to immobility in the TST [For ABX treatment, *F*(1, 13) = 11.50, *p* = 0.0048, two-way ANOVA]. **(C)** Immobility time (s) in the TST [For ABX treatment, *F*(1, 13) = 6.068, *p* = 0.0285, two-way ANOVA]. **(D)** Latency (Sec) to immobility in the FST [For ABX treatment, *F*(1, 13) = 5.950, *p* = 0.0298, two-way ANOVA]. **(E)** Immobility time (s) in the FST [For ABX treatment, *F*(1, 13) = 13.37, *p* = 0.0029, two-way ANOVA]. **(F)** The sucrose preference index (SPI) in the SPT [For ABX treatment, *F*(1, 10) = 37.09, *p* = 0.0001, two-way ANOVA]. ^**^p < 0.01. ^*^p < 0.05. N.S.: not significant. WT, *n* = 8; KO, *n* = 8; WT + ABX, *n* = 6; and KO + ABX, *n* = 6.

A flowchart of the modeling process is shown in Figure 1A. WT and Sig-1R knockout mice were divided into four groups (*n* = 8 per group): WT, WT + ABX, KO, and KO + ABX. Antibiotic treatment was conducted as described previously (Chen L. et al., 2021). Briefly, 8–9-week-old mice received a daily gavage of vancomycin (100 mg/kg), neomycin sulfate (200 mg/kg), metronidazole (200 mg/kg), and ampicillin (200 mg/kg) for 1 week. This dose of antibiotic treatment depletes the gut microbiota without causing animal death. The behavioral experiments and sample processing methods were the same as described above.

### 2.3. FMT treatment

**Figure 2:**
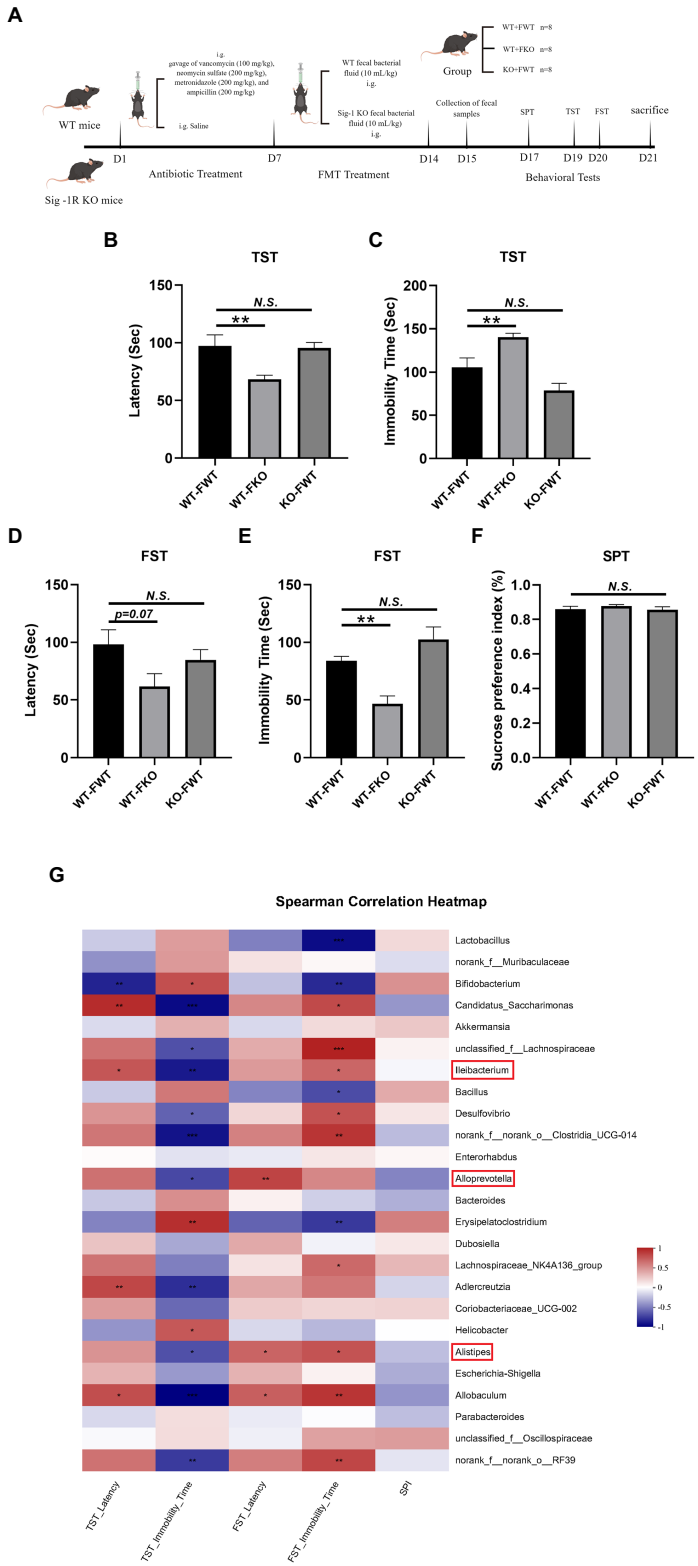
Sig-1R KO gut microbiota induced depression-like behaviors. **(A)** The FMT treatment flowchart. By Figdraw. **(B)** Latency (s) to immobility in the TST. *F*(2, 18) = 6.048, *p* = 0.0098. **(C)** Immobility time (s) in the TST. *F*(2, 18) = 14.20, *p* = 0.0002. **(D)** Latency (s) to immobility in the FST. *F*(2, 15) = 2.884, *p* = 0.0871. **(E)** Immobility time (s) in the FST. *F*(2, 15) = 13.60, *p* = 0.0004. **(F)** The sucrose preference index (SPI) in the SPT. *F*(2, 15) = 0.6173, *p* = 0.5523. ^**^p < 0.05. *N.S.*: not significant. WT-FWT, *n* = 7; WT-FKO, n = 7; and KO-FWT, *n* = 7. **(G)** The correlation between the gut microbiota and depressive-like behaviors in recipient mice was analyzed using Spearman’s correlation analysis. The red color represents a positive correlation; the blue color represents a negative correlation. Asterisk-marked columns indicated significant correlation. ^*^0.01 < *p* ≤ 0.05, ^**^0.001 < *p* ≤ 0.01, and ^***^p ≤ 0.001.

A flowchart of the modeling process is shown in Figure 2A. Fecal microbiota transplantation (FMT) was performed as previously described (Zhang et al., 2022b). Feces from WT and Sig-1R knockout mice were diluted with germ-free PBS at a ratio of 1 mg/10 μL and centrifuged (4,000 × *g*, 10 min) to separate the supernatant. Sixteen WT mice that had received antibiotic treatment as described above were randomly divided into two groups (*n* = 8 per group): WT-FWT and WT-FKO. WT fecal bacteria solution and KO fecal bacteria solution (10 mL/kg) were administered orally once daily for 1 week. In addition, eight KO mice received WT bacteria after the same pretreatment, and this group was named KO-FWT. The behavioral experiments and sample processing methods were the same as described above.

### 2.4. Tail suspension test

After 2 days of acclimatization, mice were suspended by adhesive tape on the edge of a shelf 50 cm above a tabletop. The entire experiment was recorded for 6 min using a video recorder, and the immobility of each mouse for the final 4 min was subsequently analyzed. Immobility time (in seconds) is defined as the period during which the mice stopped struggling and remained completely motionless was conducted as described previously (Steru et al., 1985).

### 2.5. Forced swimming test

After 2 days of acclimatization, mice were individually placed in circular glass chambers (diameter: 15 cm; height: 25 cm) filled with fresh water to a height of 15 cm and maintained at 25 ± 1°C. The swimming session was recorded for 6 min using a video recorder, and the immobility of each mouse for the final 4 min was subsequently analyzed (Porsolt et al., 1977).

### 2.6. Sucrose preference test

Mice were acclimated to a cage with two water bottles for 3 days. The bottle position was changed every day to prevent location preference. Twelve hours before the experiment, mice were placed in individual housing with no access to food or water. They were then given access to both 1% sucrose solution (S) and water (W). To avoid side bias, bottle positions were switched daily. The consumption of sucrose solution and water over 3 days was measured, and the sucrose preference index (SPI) of each mouse was calculated using the following formula: 100 × [VolS/(VolS + VolW)] (Zhang et al., 2017).

### 2.7. 16S rRNA gene sequencing analysis

Microbial DNA was isolated from 50 to 200 mg fecal samples using the E.Z.N.A.® tissue DNA Kit (Omega Bio-Tek). The concentration and purity of the DNA were then determined using a NanoDrop 2000 UV-vis spectrophotometer (Thermo Scientific). Primers targeting the V3-V4 regions were used to amplify the microbial DNA (338F: 5′-ACTCCTACGGGAGGCAGCAG-3′ and 806R: 5′-GGACTACHVGGGTWTCTAAT-3′). The amplified DNA was sequenced on an Illumina MiSeq platform by Majorbio Bio-Pharm Technology Co., Ltd.

The demultiplexed sequences were then merged with FLASH (v1.2.11; Magoc and Salzberg, 2011) and quality filtered with fastp (0.19.6; Chen et al., 2018). Using Qiime2 (version 2020.2) pipeline with recommended parameters, high-quality sequences were de-noised using DADA2 plugin, achieving single nucleotide resolution based on error profiles within samples. As amplicon sequence variants, DADA2 denoised sequences are usually referred to as ASVs. The number of sequence from each sample was rarefied to 4,000 in order to minimize the effect of sequencing depth on both alpha and beta diversity measures. The Naive Bayes consensus taxonomy classifier implemented in Qiime2 was used to assign the taxonomy of ASVs based on the SILVA 16S rRNA database (version 138). Data analysis was performed using the free online platform of Majorbio Cloud Platform.^1^

### 2.8. RNA sequencing

Total RNA was extracted from frozen hippocampal tissue using a QIAGEN RNeasy Mini Kit (Cat. 74,104) following homogenization in TRIzol at 4°C. The RNase-free DNase Set (79254) was used to digest and remove any residual DNA, as per the manufacturer’s instructions. RNA concentration and quality were determined using a NanoDrop with an OD of 2.0 (reference range: 1.8–2.2). All samples had RIN values >6.5.

To prepare a cDNA library for sequencing, mRNA was enriched using Oligo (dT) beads (Invitrogen) and fragmented into short RNA fragments of 300–400 nucleotides using the fragmentation buffer from the TruSeqTM RNA sample prep kit (Illumina). An oligo primer pair was used to reverse-transcribe these fragments into double-stranded cDNA (YEASEN, Shanghai, China), which was then purified using Zymo-Spin IC columns (Zymo Research, CA, United States). The purified cDNA amplicons were sequenced on an Illumina HiSeqTM 2500 following the manufacturer’s instructions (Major Biotechnology Company, Shanghai, China). Data analysis was conducted on the Majorbio Cloud platform.^2^

Using SeqPrep software^3^ and Sickle software^4^ for sequencing data quality control. The joint sequence in reads was removed, and the reads in which fragments were not inserted due to reasons such as joint self-connection were removed. The bases with low quality (less than 20) at the end of the sequence (the 3′end) are pruned away. If there is still a mass value less than 10 in the remaining sequence, the whole sequence is eliminated; otherwise, it is retained. Reads containing more than 10% *N* were removed. After discarding adapter and quality pruning sequences less than 10 bp, the original data after quality control, namely clean reads, were finally obtained. Through HiSat2^5^ for subsequent transcription of this assembly, expression of calculating mapped reads. Through RSEM,^6^ analyze the expression quantity. Using DEGseq expression differences analysis,^7^ selection criteria for FDR < 0.05 and |log2FC| ≧ 1. Follow-up analysis of the RNA sequencing data was performed using the free online platform of Majorbio Cloud Platform.^8^

### 2.9. cAMP determination

cAMP levels in hippocampal tissues were measured using a cAMP assay kit (Jiangsu LVYE Biotechnology Co., Ltd., Jiangsu, China). The assay was performed according to the manufacturer’s instructions provided in the kit.

### 2.10. Western blot

Total protein from hippocampal tissue was extracted in RIPA buffer containing protease inhibitors, and the protein concentration was determined using the CA Protein Assay Kit (Beyotime). Samples containing 10 μg of protein were separated by SDS-polyacrylamide gel electrophoresis (PAGE) and transferred to PVDF membranes. The membranes were blocked with TBST buffer containing 10% non-fat milk for 1 h at room temperature. Primary antibodies were incubated with the membranes overnight at 4°C for immunoblotting. Secondary antibodies were then incubated with the membranes for 1 h at room temperature, followed by ECL detection. The following primary antibodies were used: (a) mouse monoclonal anti-Beta Actin (1:1,000, Proteintech Group, Cat No. 66009-1-Ig); (b) rabbit monoclonal anti-BDNF (1:1,000, Abcam, Cat No. ab108319); (c) rabbit monoclonal anti-CREB1 (1:1,000, ABclonal Technology, Cat No. A10826); and (d) rabbit monoclonal anti-Phospho-CREB1-S133 (1:1,000, ABclonal Technology, Cat No. AP0019).

### 2.11. Quantitative real-time PCR analysis

Total RNA was extracted from the hippocampus of mice using TRIzol reagent (Invitrogen). The concentration and purity of the RNA were measured using a NanoDrop spectrometer. cDNA was synthesized using HifairTM II first-strand synthesis SuperMix (YEASEN). qPCR amplification was performed using HieffTM qPCR SYBR Green Master Mix (YEASEN) and the levels of mRNA expression were measured using a LightCycler® 96 System (Roche Life Science). The results were normalized to beta-actin and calculated using the CT (2^-△△CT^) method (Tan et al., 2020). A list of primers used can be found in Supplementary Table 1.

### 2.12. Statistical analysis

Statistical analysis was performed using GraphPad Prism 7.0. Quantitative results were presented as means ± SEM. Three replicates were conducted for serum biochemical analysis, western blot analysis, and RT-qPCR. One-way ANOVA with *post hoc* Tukey test and two-way ANOVA with Bonferroni posttest were used for multiple comparisons among multiple groups, and the Student’s *t* test was used for comparison between two groups. Spearman analysis was used for correlation analysis. Significance was indicated by asterisks: ^**^p < 0.05, values of *p* > 0.05 were considered not significant (N.S.).

## 3. Results

### 3.1. Antibiotic treatment reverses sig-1R knockout-induced depression-like behaviors

The ABX treatment flowchart is shown in Figure 1A. In the TST (Figure 1B), Sig-1R knockout mice exhibited a significantly shortened latency to immobility compared with WT mice, and displayed significantly increased immobility time (Figure 1C). This trend was consistent in the FST, where Sig-1R knockout mice displayed a shortened latency to immobility and prolonged immobility time (Figures 1D,E). Additionally, in the SPT, we observed a significant reduction in the sucrose preference index of the KO group compared to that of WT group (Figure 1F). Taken together, these results suggest that Sig-1R knockout elicits depression-like behaviors in mice.

To investigate the potential role of gut microbiota in Sig-1R knockout-induced depression-like behaviors, we administered an antibiotic treatment to clear the gut microbiota in mice. The results of behavioral tests indicated that this treatment eliminated the depression-like behaviors in Sig-1R knockout mice, as evidenced by the lack of significant differences in latency to immobility, immobility time, and sucrose preference index between the WT + ABX and KO + ABX groups in the TST, FST, and SPT experiments (Figures 1B–F). These findings suggest that gut microbiota may contribute to the development of Sig-1R knockout-mediated depression-like behaviors, and that antibiotic treatment can effectively eliminate these behaviors.

### 3.2. Sig-1R knockout induced dysbiosis of the gut microbiota

**Figure 3:**
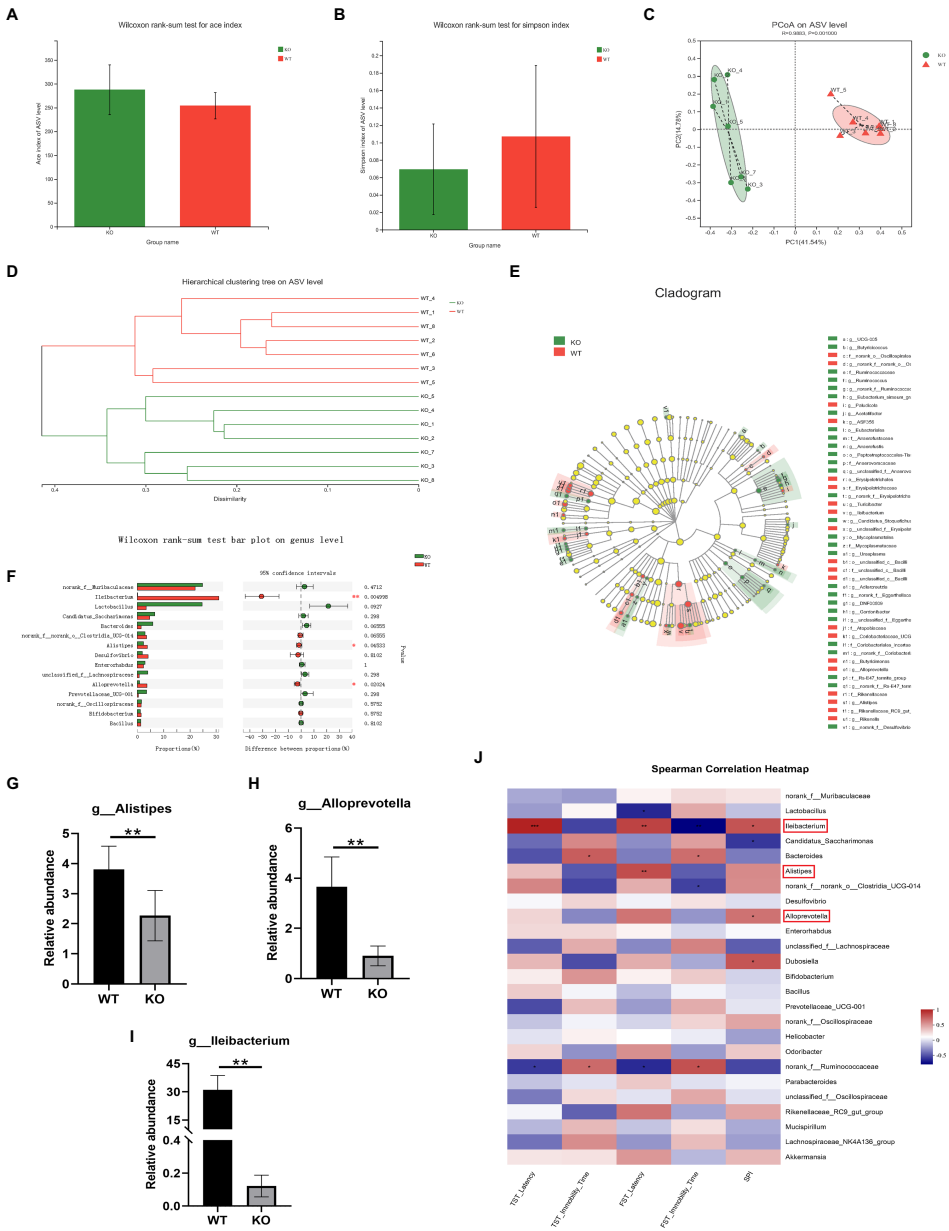
Sig-1R knockout induced dysbiosis of the gut microbiota. **(A)** ACE index and **(B)** Simpson index of the gut microbiota. **(C)** PCoA and **(D)** hierarchical clustering of 16S rRNA gene sequences. **(E)** The LEfSe analysis of the gut microbiota from the phylum level down to the genus level. **(F)** Composition of the gut microbiota at the genus level. **(G–I)** The relative abundance of *Alistipes*, *Alloprevotella*, and *Lleibacterium*. ^**^p<0.01. WT, *n* = 6; KO, *n* = 6. **(J)** The correlation between the gut microbiota and depressive-like behaviors in WT and KO mice was analyzed using Spearman’s correlation analysis. The red color represents a positive correlation; the blue color represents a negative correlation. Asterisk-marked columns indicated significant correlation. ^*^0.01 < *p* ≤ 0.05, ^**^0.001 < *p* ≤ 0.01, and ^***^p ≤ 0.001.

To examine the impact of Sig-1R knockout on gut microbiota composition, we conducted 16S rRNA sequencing and analyzed alpha diversity using the Ace index (Figure 3A) and Simpson index (Figure 3B). While we did not observe significant differences in alpha diversity between the KO and WT groups, principal coordinate analysis (PCoA) of beta diversity revealed clear differences in microbiota profiles between the two groups, with samples from each group clustering together (Figure 3C). Hierarchical clustering analysis also indicated significant differences in gut microbiota between the WT and KO groups (Figure 3D). Linear discriminant analysis effect size (LEfSe) analysis revealed significant differences in bacterial abundance at various levels of taxonomic classification, from the phylum to genus level (Figure 3E). Using the Wilcoxon rank-sum test, we further analyzed differences in gut microbiota at the genus level (Figure 3F) and found that the relative abundance of *Alistipes*, *Alloprevotella*, and *Lleibacterium* was significantly decreased in the KO group compared to the WT group (Figures 3G–I). We conducted correlation analysis to investigate the relationship between depression-like behaviors and changes in the gut microbiota, and found that the gut microbiota was closely related to depression-like behaviors (Figure 3J). These results suggest that Sig-1R knockout leads to dysbiosis of the gut microbiota.

### 3.3. FMT alters the gut microbiota in recipient mice

**Figure 4:**
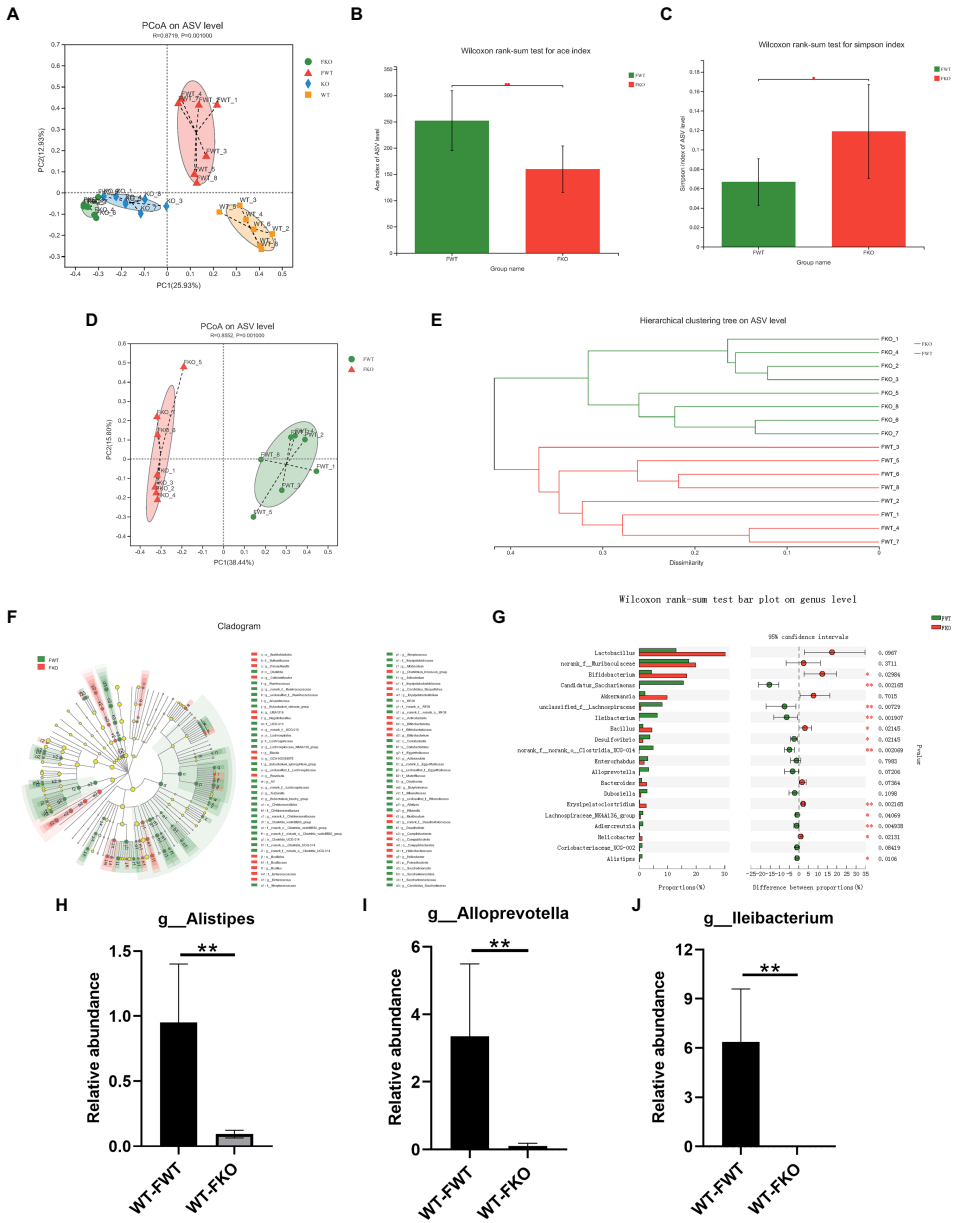
FMT alters the gut microbiota in recipient mice. **(A)** The gut microbiota PCoA analysis of four groups of mice. **(B)** ACE index and **(C)** Simpson index of the gut microbiota. **(D)** PCoA and **(E)** hierarchical clustering of 16S rRNA gene sequences. **(F)** The LEfSe analysis of the gut microbiota from the phylum level down to the genus level. **(G)** Composition of the gut microbiota at the genus level. **(H–J)** The relative abundance of *Alistipes*, *Alloprevotella*, and *Lleibacterium*. ^**^p < 0.01. WT-FWT, *n* = 6; WT-FKO, *n* = 6.

To examine the relationship between gut microbiota and depression-like behaviors, we performed an FMT experiment in which the gut microbiota of KO or WT groups were transplanted into recipient mice. The flowchart of the modeling process is shown in Figure 2A. Using 16S rRNA sequencing, we found that the microbiota of the recipient mice resembled that of their respective donor mice (Figure 4A). We then compared the alpha diversity of the WT-FWT and WT-FKO groups. The Ace index (Figure 4B) and Simpson index (Figure 4C) showed that the WT-FKO group had a significantly lower diversity and richness of bacterial species compared to the WT-FWT group. Beta diversity analysis using PCoA (Figure 4D) and hierarchical clustering (Figure 4E) revealed significant differences in the composition of the two groups, with samples from each group clustering together. LEfSe analysis showed differential gut microbiota at various levels of taxonomic classification, from the phylum to genus level (Figure 4F). Further analysis at the genus level using the Wilcoxon rank-sum test revealed significant decreases in the relative abundance of *Alistipes*, *Alloprevotella*, and *Lleibacterium* in the WT-FKO group compared to the WT-FWT group (Figures 4H–J). These findings suggest that FMT reproduces the dysbiosis of the gut microbiota observed in Sig-1R knockout mice.

### 3.4. Sig-1R KO gut microbiota induced depression-like behaviors

We performed behavioral tests on FMT recipient mice. The results of the TST and FST experiments showed that the WT-FKO group exhibited a shorter latency to immobility (Figures 2B,D) and increased immobility time (Figures 2C,E) compared to the WT-FWT group, suggesting that feces from Sig-1R knockout mice induced depression-like behaviors in the recipient mice. However, there was no significant difference in SPI between the two groups in the SPT experiment (Figure 2F). In contrast, there were no significant differences in behavioral results between the KO-FWT group and the WT-FWT group, suggesting that feces from WT mice reversed Sig-1R knockout-induced depression-like behaviors. Again, we conducted correlation analysis to investigate the relationship between depression-like behaviors and changes in the gut microbiota, and found that the gut microbiota such as *Alistipes*, *Alloprevotella*, and *Lleibacterium* still showed close correlation with depression-like behavior (Figure 2G).

### 3.5. cAMP/CREB/BDNF signaling mediated depression-like behaviors in sig-1R knockout mice

**Figure 5:**
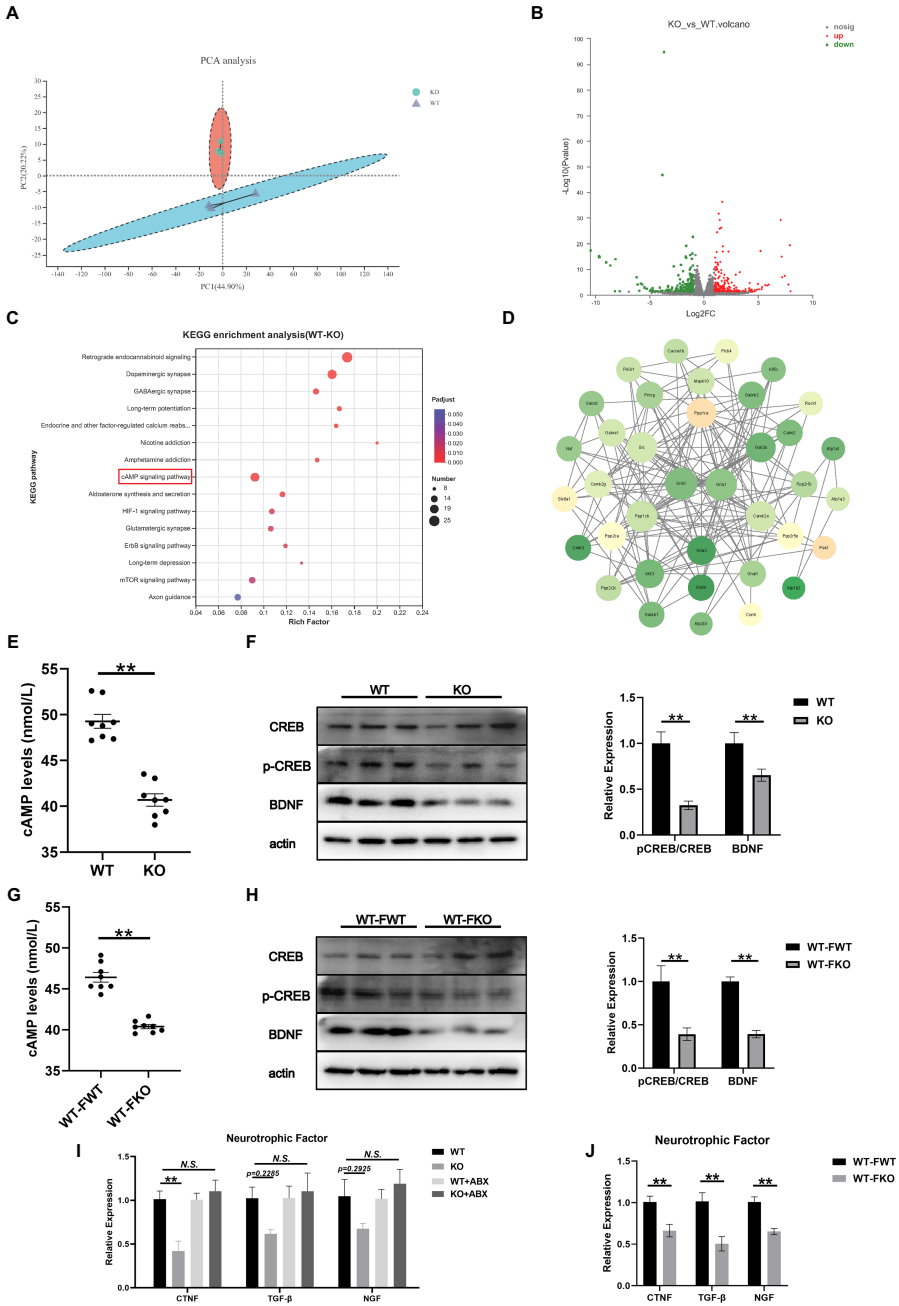
cAMP/CREB/BDNF signaling mediated depression-like behaviors in Sig-1R knockout mice. **(A)** PCA of the RNA-seq data. **(B)** Volcano plots of the DEGs from RNA-seq, with 241 upregulated and 204 downregulated. Red color intensity signifies upregulation of gene expression, and green signifies downregulation. **(C)** Mapping of the RNA-seq data to the KEGG pathway (KEGG). The vertical axis represents the pathway names, while the horizontal axis represents the enrichment factor. The size of the point indicates the number of DEGs in the pathway, and the color of the point corresponds to a different *Q*-value range. **(D)** PPI network analysis. The darker color indicates a higher core degree. **(E)** cAMP levels in WT and KO group. **(F)** Western blotting and statistical analysis of cAMP/CREB/BDNF signaling pathway in WT and KO group. **(G)** cAMP levels in FMT recipient mice. **(H)** Western blotting and statistical analysis of cAMP/CREB/BDNF signaling pathway in FMT recipient mice. **(I,J)** RT-qPCR for the expression of neurotrophic factors CTNF, TGF-β, and NGF [CTNF *F*(3, 12) = 9.073, *p* = 0.0021; TGF-β *F*(2, 15) = 2.413, *p* = 0.1174. *F*(2, 15) = 2.395, *p* = 0.1192. One-way ANOVA]. ^**^p < 0.05. *N.S.*: not significant.

To further investigate the mechanism underlying depression-like behaviors in Sig-1R knockout mice, we performed RNA sequencing on the hippocampus of WT and Sig-1R knockout mice. PCA revealed significant differences between the two groups (Figure 5A). Using a fold change threshold of 2 and a value of *p* of <0.05, a Volcano plot analysis showed that 241 mRNAs were upregulated and 204 were downregulated (Figure 5B). We conducted KEGG enrichment analysis and protein–protein interaction (PPI) analysis on the differentially expressed genes. KEGG enrichment analysis revealed that several pathways related to depression, including the retrograde endocannabinoid signaling pathway, dopaminergic synapse, GABAergic synapse, and cAMP signaling pathway, were enriched (Figure 5C). PPI analysis identified Grin1 and Gria1 as the most central genes in the PPI network; these genes have been previously linked to depression (Figure 5D).

Hippocampus-derived brain-derived neurotrophic factor (BDNF) has been identified as a significant factor in the development of depression. It is well known that the expression of BDNF is regulated by cAMP response element binding protein (CREB). In this study, we aimed to investigate the role of the cAMP/CREB/BDNF signaling pathway in Sig-1R knockout-induced depression-like behaviors. To do this, we compared the levels of cAMP and the expressions of pCREB/CREB and BDNF in the WT and KO groups. We found that the level of cAMP was significantly lower and the expression levels of pCREB/CREB and BDNF were significantly decreased in the KO group (Figures 5E,F). We also examined the cAMP/CREB/BDNF signaling pathway in FMT recipient mice and found that compared to the WT-FWT group, the level of cAMP was significantly decreased and the protein expression levels of pCREB/CREB and BDNF were significantly decreased in the WT-FKO group (Figures 5G,H). These results suggest that the cAMP/CREB/BDNF signaling pathway is inhibited in the KO group, leading to reduced expression of neurotrophic factors. Additionally, we examined the expression of three other common neurotrophic factors (CTNF, TGF-β, and NGF) in the KO and WT groups, and found that the expression of these factors showed a downward trend in the KO group compared to the WT group. However, after ABX treatment, the differential expression disappeared (Figure 5I). Similarly, WT-FKO mice receiving fecal bacteria from KO mice also showed significantly reduced expression of CTNF, TGF-β, and NGF (Figure 5J).

## 4. Discussion

Recent research has indicated that there is a close relationship between the balance of gut microbiota and depression. In this study, we found that Sig-1R knockout induced depression-like behaviors in mice, which was reversed upon clearance of gut microbiota with antibiotic treatment. Our findings were further supported by the observation that gut microbiota dysfunction and depression-like phenotypes in Sig-1R knockout mice could be reproduced through FMT experiments. Additionally, hippocampal RNA sequencing identified multiple KEGG pathways that are associated with depression. We also discovered that gut microbiota from Sig-1R-deficient mice may induce depression-like behaviors through regulation of the cAMP/CREB/BDNF signaling pathway. These results provide further evidence for the underlying mechanisms of depression and the link between the gut and the brain.

Sigma-1 receptor is highly expressed in brain regions related to mood. In our study, we found that Sig-1R knockout mice displayed a depression-like phenotype, including a significant reduction in immobility time and an increase in latency to immobility in the FST and TST, consistent with previous findings (Sabino et al., 2009). However, the mechanisms by which Sig-1R modulates antidepressant-like behaviors remain unclear. Previous studies have proposed that the serotonergic system may be involved (Szabo et al., 2021; Wu et al., 2021). Qin et al. (2022) found that inhibition of Sig-1R reduces PKC phosphorylation and suppresses GABAAR expression, leading to depression-like behaviors (Qin et al., 2022). Sha et al. (2015) also found that Sig-1R deficiency impairs neurogenesis, resulting in a depression-like phenotype (Sha et al., 2015). In our study, we found that the gut-brain axis may be a potential mechanism through which Sig-1R regulates depression-like behaviors. Sig-1R knockout mice displayed a depression-like phenotype that disappeared after depletion of gut microbiota with antibiotic treatment, and FMT could reproduce Sig-1R-mediated depression-like phenotypes. In recent years, neuroscientific research has demonstrated the importance of microbiota in depression (Evrensel and Ceylan, 2015; Chang et al., 2022). It has been reported that anxiety, depression, and aggressive behaviors can occur in mice with altered gut microbiota. FMT from various rodent models, such as unpredictable chronic mild stress (UCMS) models (Chevalier et al., 2020) and DSS models (Zhang F. et al., 2022), can reproduce depression-like phenotypes. This is the first study to confirm that feces from Sig-1R knockout mice had the same effect. Notably, no effect was observed on the SPI of the SPT after FMT treatment. We speculate that Sig-1R knockout did not cause severe depressive symptoms, which might be reflected in the SPT. Anhedonia in the sucrose preference test is defined as a preference for sucrose <65% (Scheggi et al., 2018; Fonseca-Rodrigues et al., 2022). Sig-1R knockout caused only minor variations in SPI, which may have been masked by FMT treatment.

Increasing evidence supports the connection between depression and the gut microbiota (Valles-Colomer et al., 2019; Yang et al., 2019). Depression is associated with decreased richness and diversity of the gut microbiota (Kelly et al., 2016). The pattern of the gut microbiome differs significantly between patients with depression and healthy controls (Barandouzi et al., 2020; Bertsch et al., 2022). In our study, Sig-1R knockout significantly altered the composition of the gut microbiota. At the genus level, the abundance of *Alistipes*, *Alloprevotella*, and *Lleibacterium* decreased significantly, and these changes were also observed in mice receiving FMT treatment. *Alistipes*, belonging to the family *Rikenellaceae*, has been shown to be significantly associated with depression (Jiang et al., 2015; Zhong et al., 2022). *Alistipes* has also been linked to depression-like phenotypes in other mood disorders (Domènech et al., 2022; Zhang et al., 2022a). In patients taking antidepressants, the abundance of *Alistipes* increased significantly, suggesting that it may play a crucial role in antidepressant effects (Chen Q. et al., 2021). *Alloprevotella*, which produces short-chain fatty acids and indole derivatives that may be involved in depression (Zheng et al., 2021), is more abundant in healthy patients compared to those with depression (Chen et al., 2022; Zhang K. et al., 2022). Depression-like symptoms in mice subjected to unpredictable chronic mild stress were relieved after CD36 knockout, and analysis of gut microbiota in CD36 KO mice revealed a significant increase in the abundance of *Alloprevotella* (Bai et al., 2021). A study on postpartum depression also found that *Alloprevotella* was associated with depressive phenotypes (Tian et al., 2021). A decrease in the relative abundance of *Lleibacterium*, which may be involved in enzymes that participate in purine metabolism, was observed in patients with major depression (Jiang et al., 2015). A reduced abundance of *Lleibacterium* was also found in our study. A recent study indicated that the abundance of *Lleibacterium* increased after consumption of dechlorogenic sunflower seeds and was negatively associated with depression-like phenotypes and markers of mucosal barrier damage (Lu et al., 2022).

ABX treatment has been demonstrated to alter gut microbiota composition and diversity dramatically in the host (Kennedy et al., 2018). To examine the role of gut microbiota in depression-like behaviors, ABX treatment was used to deplete gut microbiota. In our experiment, depression-like behaviors of Sig-1R KO mice were alleviated after ABX treatment, with changes in neurotrophic factors, suggesting that the disruption of gut microbiota caused by Sig-1R KO may play a significant role in the occurrence of depression-like behaviors. Although there was no statistically significant difference in immobility time in TST and latency to immobility in FST, it also showed a downward trend, which may be limited due to the restricted sample size. Consistent with our results, anhedonic-like phenotypes in chronic social defeat stress (CSDS) mice were alleviated after ABX treatment (Wang et al., 2020), suggesting that ABX-induced microbiome failure leads to stress resilience. Wang also found that antibiotic treatment alleviated depression-like behavior and decreased prefrontal cortical synaptic proteins in CSDS mice (Wang S. et al., 2021).

The vagus nerve system regulates the bidirectional communication between the gut microbiota and the brain. Extensive research has demonstrated that subdiaphragmatic vagotomy (SDV) can ameliorate depression-like behaviors. Subdiaphragmatic vagotomy block the depression-like phenotypes in ABX-treated mice with FMT from α7 subtype of the nicotinic acetylcholine receptor (Chrna7) KO mice (Pu et al., 2021). Behavior abnormalities in mice transplanted with CSDS fecal bacteria were significantly blocked by SDV (Wang et al., 2020). In our study, we found that the cAMP/CREB/BDNF signaling pathway was substantially downregulated in mice transplanted with Sig-1R KO fecal bacteria. However, the potential mechanism by which gut microbiota affects the brain remains unknown. The vagus nerve is associated with the development of neurological diseases by regulating immunity and inflammation. The association between the vagus nerve and depression-like behavior mediated by Sig-1R KO gut microbiota seems to be a research direction worth exploring.

There is a growing body of evidence linking BDNF to the pathogenesis of depression. In our study, lower levels of BDNF were also observed in Sig-1R knockout mice and mice transplanted with feces from Sig-1R knockout mice, compared to control mice. BDNF levels are significantly lower in patients with major depression than in healthy controls, and they recover after antidepressant treatment (Chen et al., 2001). This may be related to BDNF’s role in regulating synaptic plasticity and neurogenesis (Björkholm and Monteggia, 2016; Castrén and Kojima, 2017; Colucci-D’Amato et al., 2020). Additionally, we examined several other neurotrophic factors, including CTNF, TGF-β, and NGF, and found that their mRNA expression levels were also significantly reduced in Sig-1R knockout mice and FMT-KO mice. Decreased plasma levels of TGF-1 are associated with depression severity in patients with MDD (Caraci et al., 2018). Imbalances between IL-6 and TGF-β and between Th17 and Treg were also found in depressed patients or in animal models of depression, which induces neuroinflammation and neuronal dysfunction (Huang et al., 2022). These neurotrophic factors play crucial roles in memory formation and synaptic plasticity and have been identified as potential targets for the treatment of depression (Oglodek et al., 2016; Mondal and Fatima, 2019).

Brain-derived neurotrophic factor is a direct target of CREB (Barco et al., 2005), and cAMP-activated phosphorylated CREB regulates the expression of neurotrophic factors such as BDNF and NGF (Ortega-Martinez, 2015). The cAMP/CREB/BDNF pathway has been shown to be an important mechanism of action in traditional Chinese medicine (Cai et al., 2022) and novel antidepressants such as vortioxetine (Ramezany et al., 2019). In the hippocampal mRNA sequencing of Sig-1R knockout mice, the cAMP signaling pathway was significantly enriched. We also verified that the cAMP/CREB/BDNF signaling pathway was significantly downregulated at the protein level in Sig-1R knockout mice. FMT from Sig-1R knockout mice also resulted in downregulation of the cAMP/CREB/BDNF signaling pathway. This may be a potential mechanism by which the microbiome mediates depression-like behaviors. Through PPI network analysis of mRNA sequencing, we identified two core differentially expressed, Grin1 and Gria1. Genetic changes in Grin1 may increase the risk of depression (Weder et al., 2014). Nucleotide polymorphisms of Gria1 are associated with serotonin reuptake disorders in patients with depression (Bishop et al., 2012).

In our study, we found that the gut-brain axis may be a potential mechanism through which Sig-1R regulates depression-like behaviors. Our study is the first to provide evidence that fecal microbiota from Sig-1R knockout mice can induce depressive-like behavior in recipient mice. Our study offers new insights into the complex interplay between the gut and brain, specifically regarding the role of Sig-1R in regulating depressive-like behaviors.

## 5. Conclusion

**Figure 6:**
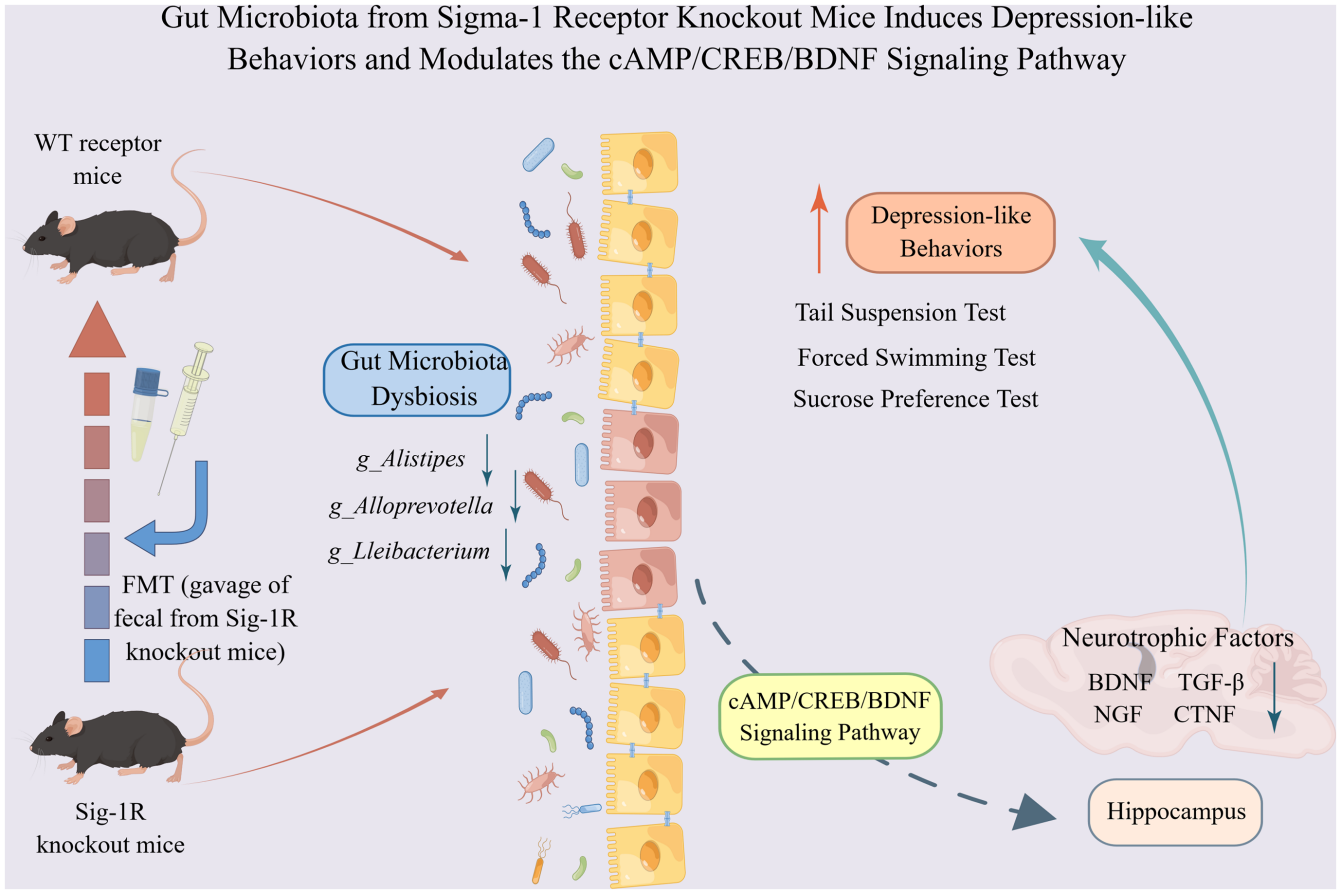
Gut microbiota from Sigma-1 receptor knockout mice induces depression-like behaviors and modulates the cAMP/CREB/BDNF signaling pathway. Sig-1R knockout induced increased depression-like behaviors in mice with decreased neurotrophic factors, which may be mediated by gut microbiota. The microbiota from Sig-1R knockout mice was sufficient to cause a depression-like phenotype and a reduction in neurotrophic factors. Gut microbiota may regulate the expressions of neurotrophic factors through the cAMP/CREB/BDNF signaling pathway. By Figdraw.

In this study, we showed that Sig-1R knockout induced increased depression-like behaviors in mice with decreased neurotrophic factors, which may be mediated by gut microbiota. The microbiota from Sig-1R knockout mice was sufficient to cause a depression-like phenotype and a reduction in neurotrophic factors. The gut microbiota may regulate the expression of neurotrophic factors through the cAMP/CREB/BDNF signaling pathway (Figure 6). Our study offers a new perspective on the mechanism of action of Sig-1R in the regulation of depression and provides further evidence for the study of the gut-brain axis.

## Data availability statement

The datasets presented in this study can be found in online repositories. The names of the repository/repositories and accession number(s) can be found at: https://ngdc.cncb.ac.cn/gsa/browse/CRA009544, CRA009544.

## Ethics statement

The animal study was reviewed and approved by SMUL20211046.

## Author contributions

J-HL performed the experiments and wrote the manuscript. J-LL contributed to the establishment of the animal model and data collection. X-WL, YL, and J-ZY contributed to data collection and analysis. L-JC and K-KZ participated in manuscript revision and provided constructive suggestions. X-LX and QW contributed to the conception and design of the study and provided financial support for the research leading to this publication. All authors contributed to the article and approved the submitted version.

## Funding

This work was supported by the National Natural Science Foundation of China under Grant No. 82171877; the Guangdong Natural Science Foundation under Grant No. 2021A1515012456; and the Department of Science and Technology of Guangzhou city under Grant No. 202002030043.

## Conflict of interest

The authors declare that the research was conducted in the absence of any commercial or financial relationships that could be construed as a potential conflict of interest.

## Publisher’s note

All claims expressed in this article are solely those of the authors and do not necessarily represent those of their affiliated organizations, or those of the publisher, the editors and the reviewers. Any product that may be evaluated in this article, or claim that may be made by its manufacturer, is not guaranteed or endorsed by the publisher.

## Footnotes

^1^ cloud.majorbio.com

^2^ www.majorbio.com

^3^ https://github.com/jstjohn/SeqPrep

^4^ https://github.com/najoshi/sickle

^5^ http://ccb.jhu.edu/software/hisat2/index.shtml

^6^ http://deweylab.github.io/RSEM/

^7^ https://www.rdocumentation.org/packages/DEGseq/versions/1.26.0

^8^ www.majorbio.com

## Supplementary material

SUPPLEMENTARY FIGURE S1 PPCR rewsults of Sig-1R KO mice genotype identification.

## References

[ref1] Aizawa E. Tsuji H. Asahara T. Takahashi T. Teraishi T. Yoshida S.. (2016). Possible association of bifidobacterium and lactobacillus in the gut microbiota of patients with major depressive disorder. J. Affect. Disord. 202, 254–257. doi: 10.1016/j.jad.2016.05.038, PMID: 27288567

[ref2] Bai S. Wang W. Wang T. Li J. Zhang S. Chen Z.. (2021). Cd36 deficiency affects depressive-like behaviors possibly by modifying gut microbiota and the inflammasome pathway in mice. Transl. Psychiatry 11:16. doi: 10.1038/s41398-020-01130-8, PMID: 33414380 PMC7791141

[ref3] Barandouzi Z. A. Starkweather A. R. Henderson W. A. Gyamfi A. Cong X. S. (2020). Altered composition of gut microbiota in depression: a systematic review. Front. Psychol. 11:541. doi: 10.3389/fpsyt.2020.00541 32587537 PMC7299157

[ref4] Barco A. Patterson S. L. Alarcon J. M. Gromova P. Mata-Roig M. Morozov A.. (2005). Gene expression profiling of facilitated l-ltp in vp16-creb mice reveals that bdnf is critical for the maintenance of ltp and its synaptic capture. Neuron 48, 123–137. doi: 10.1016/j.neuron.2005.09.005, PMID: 16202713

[ref5] Bertsch I. Courtois R. Gallard E. Reveillere C. Pham T. H. (2022). Is it possible to use the hcr-20 v2 to assess the risk of violent recidivism of french offenders? Foren. Sci. Res. 7, 402–411. doi: 10.1080/20961790.2022.2046370, PMID: 36353320 PMC9639531

[ref6] Bishop J. R. Chae S. S. Patel S. Moline J. Ellingrod V. L. (2012). Pharmacogenetics of glutamate system genes and ssri-associated sexual dysfunction. Psychiatry Res. 199, 74–76. doi: 10.1016/j.psychres.2012.03.048, PMID: 22534499 PMC3458139

[ref7] Björkholm C. Monteggia L. M. (2016). Bdnf—a key transducer of antidepressant effects. Neuropharmacology 102, 72–79. doi: 10.1016/j.neuropharm.2015.10.034, PMID: 26519901 PMC4763983

[ref8] Cai M. Y. Yang Z. Huang X. J. Li J. Bao W. Y. Hurilebagen. (2022). Mongolian medicine areca thirteen pill (gy-13) improved depressive syndrome via upregulating camp/pka/creb/bdnf signaling pathway. J. Ethnopharmacol. 293:115310. doi: 10.1016/j.jep.2022.115310, PMID: 35452773

[ref9] Caraci F. Spampinato S. F. Morgese M. G. Tascedda F. Salluzzo M. G. Giambirtone M. C.. (2018). Neurobiological links between depression and ad: the role of tgf-β1 signaling as a new pharmacological target. Pharmacol. Res. 130, 374–384. doi: 10.1016/j.phrs.2018.02.007, PMID: 29438781

[ref10] Castrén E. Kojima M. (2017). Brain-derived neurotrophic factor in mood disorders and antidepressant treatments. Neurobiol. Dis. 97, 119–126. doi: 10.1016/j.nbd.2016.07.010 27425886

[ref11] Castren E. Rantamaki T. (2010). The role of bdnf and its receptors in depression and antidepressant drug action: reactivation of developmental plasticity. Dev. Neurobiol. 70, 289–297. doi: 10.1002/dneu.20758 20186711

[ref12] Chang L. Wei Y. Hashimoto K. (2022). Brain–gut–microbiota axis in depression: a historical overview and future directions. Brain Res. Bull. 182, 44–56. doi: 10.1016/j.brainresbull.2022.02.004, PMID: 35151796

[ref13] Chen B. Dowlatshahi D. MacQueen G. M. Wang J. Young L. T. (2001). Increased hippocampal bdnf immunoreactivity in subjects treated with antidepressant medication. Biol. Psychiatry 50, 260–265. doi: 10.1016/S0006-3223(01)01083-6 11522260

[ref14] Chen L. He J. Pan M. Liu J. Zhang K. Li J.. (2021). Antibiotics attenuate methamphetamine-induced hepatotoxicity by regulating oxidative stress and tlr4/myd88/traf6 axis. Front. Pharmacol. 12:716703. doi: 10.3389/fphar.2021.716703, PMID: 34381368 PMC8350338

[ref15] Chen Q. He Z. Zhuo Y. Li S. Yang W. Hu L.. (2021). Rubidium chloride modulated the fecal microbiota community in mice. BMC Microbiol. 21:46. doi: 10.1186/s12866-021-02095-4, PMID: 33588762 PMC7885239

[ref16] Chen Y. Tian P. Wang Z. Pan R. Shang K. Wang G.. (2022). Indole acetic acid exerts anti-depressive effects on an animal model of chronic mild stress. Nutrients 14:5019. doi: 10.3390/nu14235019, PMID: 36501051 PMC9737131

[ref17] Chen S. Zhou Y. Chen Y. Gu J. (2018). Fastp: an ultra-fast all-in-one fastq preprocessor. Bioinformatics 34, i884–i890. doi: 10.1093/bioinformatics/bty560, PMID: 30423086 PMC6129281

[ref18] Chevalier G. Siopi E. Guenin-Macé L. Pascal M. Laval T. Rifflet A.. (2020). Effect of gut microbiota on depressive-like behaviors in mice is mediated by the endocannabinoid system. Nat. Commun. 11:6363. doi: 10.1038/s41467-020-19931-2, PMID: 33311466 PMC7732982

[ref19] Colucci-D’Amato L. Speranza L. Volpicelli F. (2020). Neurotrophic factor bdnf, physiological functions and therapeutic potential in depression, neurodegeneration and brain cancer. Int. J. Mol. Sci. 21:7777. doi: 10.3390/ijms21207777, PMID: 33096634 PMC7589016

[ref20] Domènech L. Willis J. Alemany-Navarro M. Morell M. Real E. Escaramís G.. (2022). Changes in the stool and oropharyngeal microbiome in obsessive-compulsive disorder. Sci. Rep. 12:1448. doi: 10.1038/s41598-022-05480-9, PMID: 35087123 PMC8795436

[ref21] Duman R. S. Monteggia L. M. (2006). A neurotrophic model for stress-related mood disorders. Biol. Psychiatry 59, 1116–1127. doi: 10.1016/j.biopsych.2006.02.013 16631126

[ref22] Evrensel A. Ceylan M. E. (2015). The gut-brain axis: the missing link in depression. Clin. Psychopharmacol. Neurosci. 13, 239–244. doi: 10.9758/cpn.2015.13.3.239, PMID: 26598580 PMC4662178

[ref23] Fehér Á. Juhász A. László A. Kálmán J. Jr. Pákáski M. Kálmán J.. (2012). Association between a variant of the sigma-1 receptor gene and Alzheimer’s disease. Neurosci. Lett. 517, 136–139. doi: 10.1016/j.neulet.2012.04.046, PMID: 22561649

[ref24] Fonseca-Rodrigues D. Gonçalves J. Laranjeira I. Almeida A. Pinto-Ribeiro F. (2022). Sucrose intake and preference by wistar han rats are not influenced by sex or food/water deprivation. Pharmacol. Biochem. Behav. 216:173387. doi: 10.1016/j.pbb.2022.173387 35429511

[ref25] Fujimoto M. Hayashi T. Urfer R. Mita S. Su T. (2012). Sigma-1 receptor chaperones regulate the secretion of brain-derived neurotrophic factor. Synapse 66, 630–639. doi: 10.1002/syn.21549, PMID: 22337473 PMC3824965

[ref26] Fujimoto T. Takeuchi K. Matsumoto T. Fujita S. Honda K. Higashi Y.. (2008). Metabolic changes in the brain of patients with late-onset major depression. Psychiatry Res. 164, 48–57. doi: 10.1016/j.pscychresns.2007.03.010, PMID: 18804352

[ref27] Hashimoto K. (2009). Sigma-1 receptors and selective serotonin reuptake inhibitors: clinical implications of their relationship. Cent. Nerv. Syst. Agents Med. Chem. 9, 197–204. doi: 10.2174/1871524910909030197 20021354

[ref28] Hashimoto K. (2010). Brain-derived neurotrophic factor as a biomarker for mood disorders: an historical overview and future directions. Psychiatry Clin. Neurosci. 64, 341–357. doi: 10.1111/j.1440-1819.2010.02113.x 20653908

[ref29] Hashimoto K. (2013). Sigma-1 receptor chaperone and brain-derived neurotrophic factor: emerging links between cardiovascular disease and depression. Prog. Neurobiol. 100, 15–29. doi: 10.1016/j.pneurobio.2012.09.001, PMID: 23044468

[ref30] Huang C. Zhang F. Li P. Song C. (2022). Low-dose il-2 attenuated depression-like behaviors and pathological changes through restoring the balances between il-6 and tgf-β and between th17 and treg in a chronic stress-induced mouse model of depression. Int. J. Mol. Sci. 23:13856. doi: 10.3390/ijms232213856, PMID: 36430328 PMC9699071

[ref31] Izumi Y. Reiersen A. M. Lenze E. J. Mennerick S. J. Zorumski C. F. (2023). Ssris differentially modulate the effects of pro-inflammatory stimulation on hippocampal plasticity and memory via sigma 1 receptors and neurosteroids. Transl. Psychiatry 13:39. doi: 10.1038/s41398-023-02343-3, PMID: 36737431 PMC9897619

[ref32] Ji L. Peng J. Fu C. Tong L. Wang Z. (2017). Sigma-1 receptor activation ameliorates anxiety-like behavior through nr2a-creb-bdnf signaling pathway in a rat model submitted to single-prolonged stress. Mol. Med. Rep. 16, 4987–4993. doi: 10.3892/mmr.2017.7185, PMID: 28791385

[ref33] Jiang H. Ling Z. Zhang Y. Mao H. Ma Z. Yin Y.. (2015). Altered fecal microbiota composition in patients with major depressive disorder. Brain Behav. Immun. 48, 186–194. doi: 10.1016/j.bbi.2015.03.016 25882912

[ref34] Kelly J. R. Borre Y. O’ Brien C. Patterson E. el Aidy S. Deane J.. (2016). Transferring the blues: depression-associated gut microbiota induces neurobehavioural changes in the rat. J. Psychiatr. Res. 82, 109–118. doi: 10.1016/j.jpsychires.2016.07.019, PMID: 27491067

[ref35] Kennedy E. A. King K. Y. Baldridge M. T. (2018). Mouse microbiota models: comparing germ-free mice and antibiotics treatment as tools for modifying gut bacteria. Front. Physiol. 9:1534. doi: 10.3389/fphys.2018.01534, PMID: 30429801 PMC6220354

[ref36] Kishi T. Yoshimura R. Okochi T. Fukuo Y. Kitajima T. Okumura T.. (2010). Association analysis of sigmar1 with major depressive disorder and ssri response. Neuropharmacology 58, 1168–1173. doi: 10.1016/j.neuropharm.2010.02.013, PMID: 20178807

[ref37] Li N. Wang Q. Wang Y. Sun A. Lin Y. Jin Y.. (2019). Fecal microbiota transplantation from chronic unpredictable mild stress mice donors affects anxiety-like and depression-like behavior in recipient mice via the gut microbiota-inflammation-brain axis. Stress 22, 592–602. doi: 10.1080/10253890.2019.1617267, PMID: 31124390

[ref38] López-Figueroa A. L. Norton C. S. López-Figueroa M. O. Armellini-Dodel D. Burke S. Akil H.. (2004). Serotonin 5-ht1a, 5-ht1b, and 5-ht2a receptor mrna expression in subjects with major depression, bipolar disorder, and schizophrenia. Biol. Psychiatry 55, 225–233. doi: 10.1016/j.biopsych.2003.09.017, PMID: 14744462

[ref39] Lu X. Qi C. Zheng J. Sun M. Jin L. Sun J. (2022). The antidepressant effect of deoiled sunflower seeds on chronic unpredictable mild stress in mice through regulation of microbiota–gut–brain axis. Front. Nutr. 9:908297. doi: 10.3389/fnut.2022.908297, PMID: 35859751 PMC9289741

[ref40] Maes M. (2008). The cytokine hypothesis of depression: inflammation, oxidative & nitrosative stress (io&ns) and leaky gut as new targets for adjunctive treatments in depression. Neuroendocrinol. Lett. 29, 287–291. 18580840

[ref41] Magoc T. Salzberg S. L. (2011). Flash: fast length adjustment of short reads to improve genome assemblies. Bioinformatics 27, 2957–2963. doi: 10.1093/bioinformatics/btr507, PMID: 21903629 PMC3198573

[ref42] Malhi G. S. Mann J. J. (2018). Depression. Lancet 392, 2299–2312. doi: 10.1016/S0140-6736(18)31948-2 30396512

[ref43] Mandelli L. Wang S. M. Han C. Lee S. J. Patkar A. A. Masand P. S.. (2017). The impact of a single nucleotide polymorphism in sigmar1 on depressive symptoms in major depressive disorder and bipolar disorder. Adv. Ther. 34, 713–724. doi: 10.1007/s12325-017-0482-2, PMID: 28144920

[ref44] Mondal A. C. Fatima M. (2019). Direct and indirect evidences of bdnf and ngf as key modulators in depression: role of antidepressants treatment. Int. J. Neurosci. 129, 283–296. doi: 10.1080/00207454.2018.1527328, PMID: 30235967

[ref45] Oglodek E. A. Just M. J. Szromek A. R. Araszkiewicz A. (2016). Melatonin and neurotrophins nt-3, bdnf, ngf in patients with varying levels of depression severity. Pharmacol. Rep. 68, 945–951. doi: 10.1016/j.pharep.2016.04.003, PMID: 27367919

[ref46] Ortega-Martinez S. (2015). A new perspective on the role of the creb family of transcription factors in memory consolidation via adult hippocampal neurogenesis. Front. Mol. Neurosci. 8:46. doi: 10.3389/fnmol.2015.00046, PMID: 26379491 PMC4549561

[ref47] Porsolt R. D. Le Pichon M. Jalfre M. (1977). Depression: a new animal model sensitive to antidepressant treatments. Nature 266, 730–732. doi: 10.1038/266730a0 559941

[ref48] Pu Y. Tan Y. Qu Y. Chang L. Wang S. Wei Y.. (2021). A role of the subdiaphragmatic vagus nerve in depression-like phenotypes in mice after fecal microbiota transplantation from chrna7 knock-out mice with depression-like phenotypes. Brain Behav. Immun. 94, 318–326. doi: 10.1016/j.bbi.2020.12.032, PMID: 33422641

[ref49] Qin Y. Xu W. Li K. Luo Q. Chen X. Wang Y.. (2022). Repeated inhibition of sigma-1 receptor suppresses Gabaa receptor expression and long-term depression in the nucleus accumbens leading to depressive-like behaviors. Front. Mol. Neurosci. 15:959224. doi: 10.3389/fnmol.2022.959224, PMID: 36245919 PMC9563353

[ref50] Ramezany Y. S. Nourhashemi M. Keshavarzi S. Motaghinejad M. Motevalian M. (2019). Possible role of cyclic amp response element binding/brain-derived neurotrophic factor signaling pathway in mediating the pharmacological effects of duloxetine against methamphetamine use-induced cognitive impairment and withdrawal-induced anxiety and depression in rats. Adv. Biomed. Res. 8:11. doi: 10.4103/abr.abr_34_18, PMID: 30993081 PMC6425746

[ref51] Sabino V. Cottone P. Parylak S. L. Steardo L. Zorrilla E. P. (2009). Sigma-1 receptor knockout mice display a depressive-like phenotype. Behav. Brain Res. 198, 472–476. doi: 10.1016/j.bbr.2008.11.036, PMID: 19100292 PMC2667953

[ref52] Scheggi S. De Montis M. G. Gambarana C. (2018). Making sense of rodent models of anhedonia. Int. J. Neuropsychopharmacol. 21, 1049–1065. doi: 10.1093/ijnp/pyy083, PMID: 30239762 PMC6209858

[ref53] Sha S. Hong J. Qu W. Lu Z. Li L. Yu W.. (2015). Sex-related neurogenesis decrease in hippocampal dentate gyrus with depressive-like behaviors in sigma-1 receptor knockout mice. Eur. Neuropsychopharmacol. 25, 1275–1286. doi: 10.1016/j.euroneuro.2015.04.021, PMID: 25983018

[ref54] Smith S. B. Su T. P. (2017). Sigma-1 Receptor Agonists and Their Clinical Implications in Neuropsychiatric Disorders. Switzerland: Springer International Publishing AG, 153–161 10.1007/978-3-319-50174-1_11 28315270

[ref55] Steru L. Chermat R. Thierry B. Simon P. (1985). The tail suspension test: a new method for screening antidepressants in mice. Psychopharmacology 85, 367–370. doi: 10.1007/BF00428203 3923523

[ref56] Stiernstromer E. Vafors F. M. Mellgren C. Khoshnood A. (2022). Demographic characteristics of convicted child sexual abusers in south of Sweden, between 2013 and 2018: a pilot study. Foren. Sci. Res. 7, 393–401. doi: 10.1080/20961790.2022.2052590, PMID: 36353331 PMC9639549

[ref57] Szabo I. Varga V. E. Dvoracsko S. Farkas A. E. Kormoczi T. Berkecz R.. (2021). N,n-dimethyltryptamine attenuates spreading depolarization and restrains neurodegeneration by sigma-1 receptor activation in the ischemic rat brain. Neuropharmacology 192:108612. doi: 10.1016/j.neuropharm.2021.108612, PMID: 34023338

[ref58] Tan X. Zhang K. Xu J. Qu D. Chen L. Li J.. (2020). Luteolin alleviates methamphetamine-induced neurotoxicity by suppressing pi3k/akt pathway-modulated apoptosis and autophagy in rats. Food Chem. Toxicol. 137:111179. doi: 10.1016/j.fct.2020.111179, PMID: 32035215

[ref59] Tian X. Xing J. Zheng Q. Gao P. (2021). 919 syrup alleviates postpartum depression by modulating the structure and metabolism of gut microbes and affecting the function of the hippocampal gaba/glutamate system. Front. Cell. Infect. Microbiol. 11:694443. doi: 10.3389/fcimb.2021.694443, PMID: 34490139 PMC8417790

[ref60] Valles-Colomer M. Falony G. Darzi Y. Tigchelaar E. F. Wang J. Tito R. Y.. (2019). The neuroactive potential of the human gut microbiota in quality of life and depression. Nat. Microbiol. 4, 623–632. doi: 10.1038/s41564-018-0337-x, PMID: 30718848

[ref61] Wang S. Ishima T. Qu Y. Shan J. Chang L. Wei Y.. (2021). Ingestion of faecalibaculum rodentium causes depression-like phenotypes in resilient ephx2 knock-out mice: a role of brain-gut-microbiota axis via the subdiaphragmatic vagus nerve. J. Affect. Disord. 292, 565–573. doi: 10.1016/j.jad.2021.06.006, PMID: 34147969 PMC8282729

[ref62] Wang S. Ishima T. Zhang J. Qu Y. Chang L. Pu Y.. (2020). Ingestion of lactobacillus intestinalis and lactobacillus reuteri causes depression- and anhedonia-like phenotypes in antibiotic-treated mice via the vagus nerve. J. Neuroinflammation 17:241. doi: 10.1186/s12974-020-01916-z, PMID: 32799901 PMC7429467

[ref63] Wang A. Mi L. Zhang Z. Hu M. Zhao Z. Liu B.. (2021). Saikosaponin a improved depression-like behavior and inhibited hippocampal neuronal apoptosis after cerebral ischemia through p-creb/bdnf pathway. Behav. Brain Res. 403:113138. doi: 10.1016/j.bbr.2021.113138, PMID: 33493495

[ref64] Weder N. Zhang H. Jensen K. Yang B. Z. Simen A. Jackowski A.. (2014). Child abuse, depression, and methylation in genes involved with stress, neural plasticity, and brain circuitry. J. Am. Acad. Child Adolesc. Psychiatry 53, 417–424.e5. doi: 10.1016/j.jaac.2013.12.025, PMID: 24655651 PMC4126411

[ref65] Wu N. H. Ye Y. Wan B. B. Yu Y. D. Liu C. Chen Q. J. (2021). Emerging benefits: pathophysiological functions and target drugs of the sigma-1 receptor in neurodegenerative diseases. Mol. Neurobiol. 58, 5649–5666. doi: 10.1007/s12035-021-02524-5 34383254

[ref66] Yamaguchi K. Shioda N. Yabuki Y. Zhang C. Han F. Fukunaga K. (2018). Sa4503, a potent sigma-1 receptor ligand, ameliorates synaptic abnormalities and cognitive dysfunction in a mouse model of atr-x syndrome. Int. J. Mol. Sci. 19:2811. doi: 10.3390/ijms19092811, PMID: 30231518 PMC6163584

[ref67] Yang C. Fang X. Zhan G. Huang N. Li S. Bi J.. (2019). Key role of gut microbiota in anhedonia-like phenotype in rodents with neuropathic pain. Transl. Psychiatry 9:57. doi: 10.1038/s41398-019-0379-8, PMID: 30705252 PMC6355832

[ref68] Zhang K. K. Chen L. J. Li J. H. Liu J. L. Wang L. B. Xu L. L.. (2022a). Methamphetamine disturbs gut homeostasis and reshapes serum metabolome, inducing neurotoxicity and abnormal behaviors in mice. Front. Microbiol. 13:755189. doi: 10.3389/fmicb.2022.755189, PMID: 35509309 PMC9058162

[ref69] Zhang K. Cheng M. Xu J. Chen L. Li J. Li Q.. (2022). Mir-711 and mir-183-3p as potential markers for vital reaction of burned skin. Foren. Sci. Res. 7, 503–509. doi: 10.1080/20961790.2020.1719454, PMID: 36353316 PMC9639510

[ref70] Zhang K. K. Liu J. L. Chen L. J. Li J. H. Yang J. Z. Xu L. L.. (2022b). Gut microbiota mediates methamphetamine-induced hepatic inflammation via the impairment of bile acid homeostasis. Food Chem. Toxicol. 166:113208. doi: 10.1016/j.fct.2022.113208, PMID: 35688268

[ref71] Zhang J. Xie X. Tang M. Zhang J. Zhang B. Zhao Q.. (2017). Salvianolic acid b promotes microglial m2-polarization and rescues neurogenesis in stress-exposed mice. Brain Behav. Immun. 66, 111–124. doi: 10.1016/j.bbi.2017.07.012, PMID: 28736034

[ref72] Zhang F. Zhou Y. Chen H. Jiang H. Zhou F. Lv B.. (2022). Curcumin alleviates dss-induced anxiety-like behaviors via the microbial-brain-gut axis. Oxidative Med. Cell. Longev. 2022, 1–19. doi: 10.1155/2022/6244757, PMID: 35345829 PMC8957039

[ref73] Zheng P. Zeng B. Zhou C. Liu M. Fang Z. Xu X.. (2016). Gut microbiome remodeling induces depressive-like behaviors through a pathway mediated by the host’s metabolism. Mol. Psychiatry 21, 786–796. doi: 10.1038/mp.2016.44, PMID: 27067014

[ref74] Zheng S. Zhu Y. Wu W. Zhang Q. Wang Y. Wang Z.. (2021). A correlation study of intestinal microflora and first-episode depression in chinese patients and healthy volunteers. Brain Behav. 11:e02036. doi: 10.1002/brb3.2036 33960717 PMC8413750

[ref75] Zhong Q. Chen J. Wang Y. Shao W. Zhou C. Xie P. (2022). Differential gut microbiota compositions related with the severity of major depressive disorder. Front. Cell. Infect. Microbiol. 12:907239. doi: 10.3389/fcimb.2022.907239, PMID: 35899051 PMC9309346

