# Supplementary figures and images for: Gut microbiota from sigma-1 receptor knockout mice induces depression-like behaviors and modulates the cAMP/CREB/BDNF signaling pathway
Source: Front Microbiol. 2023 Apr 6;14:1143648. doi: 10.3389/fmicb.2023.1143648 (PMC10116000; doi:10.3389/fmicb.2023.1143648)

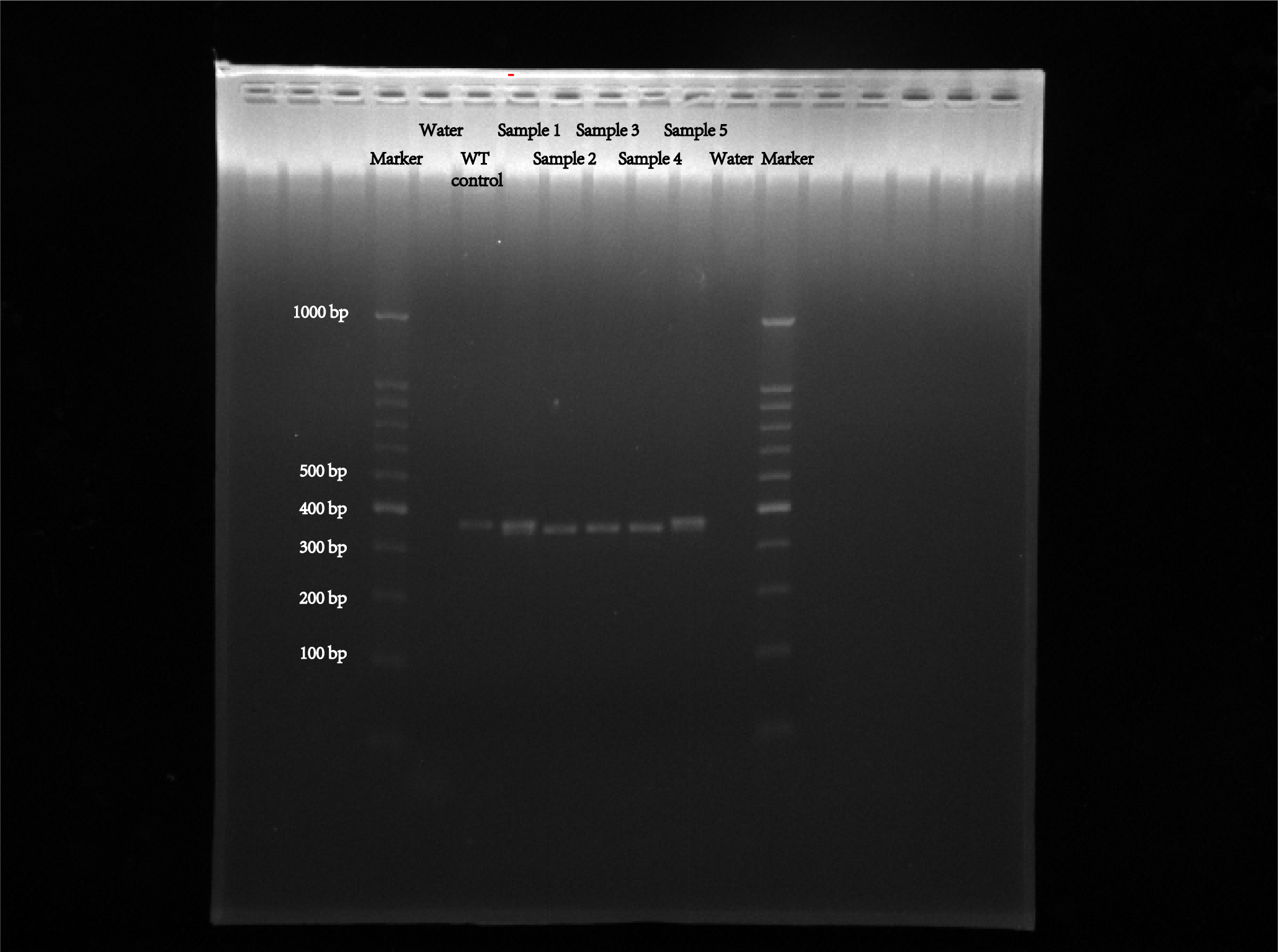

Supplement: SUPPLEMENTARY FIGURE S1 — PPCR rewsults of Sig-1R KO mice genotype identification. [file Image_1.TIF]
