# Supplementary material for: Gut microbiota from sigma-1 receptor knockout mice induces depression-like behaviors and modulates the cAMP/CREB/BDNF signaling pathway
Source: Front Microbiol. 2023 Apr 6;14:1143648. doi: 10.3389/fmicb.2023.1143648 (PMC10116000; doi:10.3389/fmicb.2023.1143648)
Supplement: Supplementary file 2 [file Table_1.DOCX]

| Gene Symbol | Forward/Reverse | Primer（5‘ to 3’） |
| --- | --- | --- |
| CTNF | Forward | TCTGTAGCCGCTCTATCTGG |
|  | Reverse | GGTACACCATCCACTGAGTCAA |
| *TGF-β* | Forward | CCACCTGCAAGACCATCGAC |
|  | Reverse | CTGGCGAGCCTTAGTTTGGAC |
| NGF | Forward | CCAGTGAAATTAGGCTCCCTG |
|  | Reverse | CCTTGGCAAAACCTTTATTGGG |
| *Actin* | Forward | CGTTGACATCCGTAAAGACC |
|  | Reverse | ACAACAGTCCGCCTAGAAGC |
| SIG KO PCR Primers | Forward | TTCTGTGCTAGCAGACCTAGAAAG |
|  | Reverse | GCTGTTTAGACACATAAGGAAACGA |
| WT PCR Primers | Forward | TTCTGTGCTAGCAGACCTAGAAAG |
|  | Reverse | AGAGAAGACGAAGTTTTGAGTGCC |

**Supplementary Table 1. The primers used in the current study.**
